# Supplementary material for: Identification of non-charged 7.44 analogs interacting with the NHR2 domain of RUNX1-ETO with improved antiproliferative effect in RUNX-ETO positive cells
Source: Sci Rep. 2025 May 22;15:17720. doi: 10.1038/s41598-025-02422-z (PMC12095692; doi:10.1038/s41598-025-02422-z)
Supplement: Supplementary file 1 — Supplementary Material 1. [file 41598_2025_2422_MOESM1_ESM.pdf]

Supporting Information

Identification of non-charged 7.44 analogs interacting with the NHR2 domain of RUNX1-ETO with improved antiproliferative effect in RUNX-ETO positive cells

Mohanraj Gopalswamy [1], David Bickel [1,2,3], Niklas Dienstbier [4], Jia-Wey Tu [4], Melina Vogt [4], Stephan Schott-Verdugo [1,6], Sanil Bhatia [4], and Manuel Etzkorn [5], Holger Gohlke [1,6,*]

1 Institute for Pharmaceutical and Medicinal Chemistry, Heinrich Heine University Düsseldorf, Düsseldorf, Germany

2 Interuniversity Institute of Bioinformatics in Brussels, Brussels, Belgium

3 Structural Biology Brussels, Vrije Universiteit Brussels, Brussels, Belgium

4 Department of Pediatric Oncology, Hematology and Clinical Immunology, Medical Faculty, Heinrich Heine University Düsseldorf, Düsseldorf, Germany

5 Institute for Physical Biology, Heinrich Heine University Düsseldorf, Universitätsstr. 1, 40225, Düsseldorf, Germany

6 Institute of Bio- and Geosciences (IBG-4: Bioinformatics), Forschungszentrum Jülich GmbH, Jülich, Germany

Supplementary figures

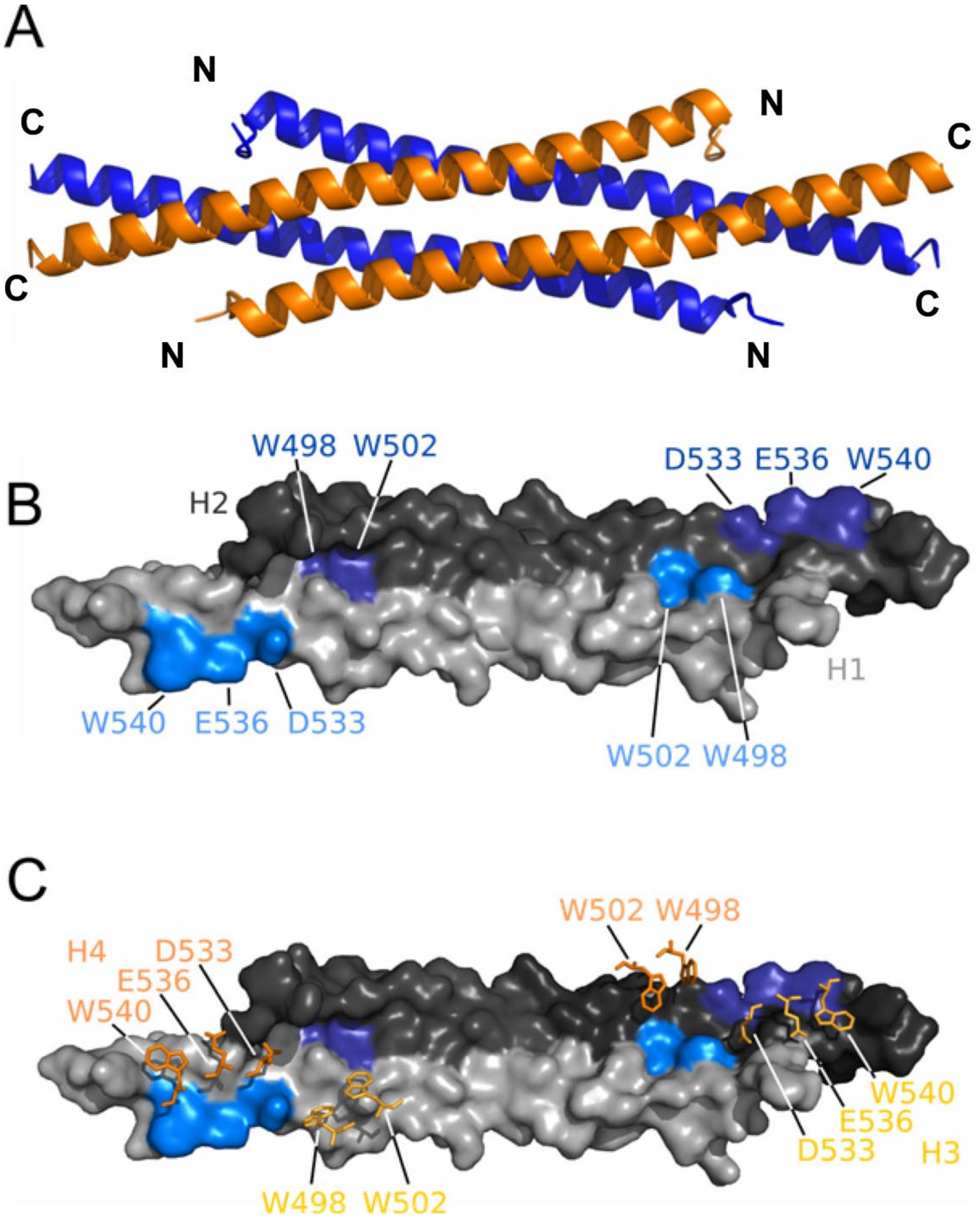

Figure S1. Structure and hot spots of NHR2. (A) NHR2 tetramer formed as a dimer of dimers, blue: helix 1 of NHR2 (H1) and helix 2 of NHR2 (H2), orange: helix 3 of NHR2 (H3) and helix 4 of NHR2 (H4). (B) Hot Spots (W498, W502, D533, E536, and W540) of an NHR2 dimer, light grey: surface of H1, dark grey: surface of H2, light blue: hot spots on H1 (surface), dark blue: hot spots on H2 (surface). (C) Representation of the organization of hot spots in the interaction interface of the NHR2 tetramer, in addition to panel C: dark orange: hot spots of H3, light orange: hot spots of H4.

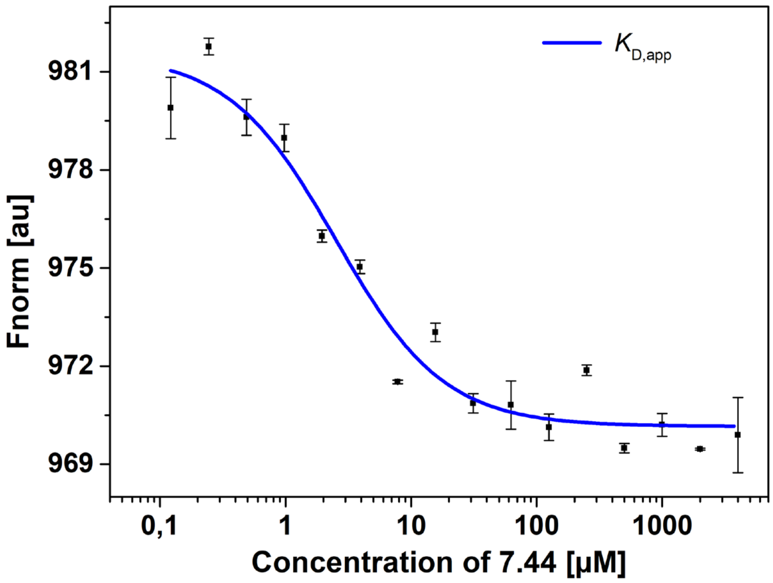

Figure S2. 7.44 interaction with NHR2 studied by MST assay. Titration of **7.44** compounds to a constant concentration of Alexa488 dye-labeled NHR2 induces a change in thermophoresis. The data previously obtained in ref. (1) were fitted to a 1:1 binding model to obtain an apparent dissociation constant $K_{D,app}$ of 2.3 ± 0.7 µM (blue).

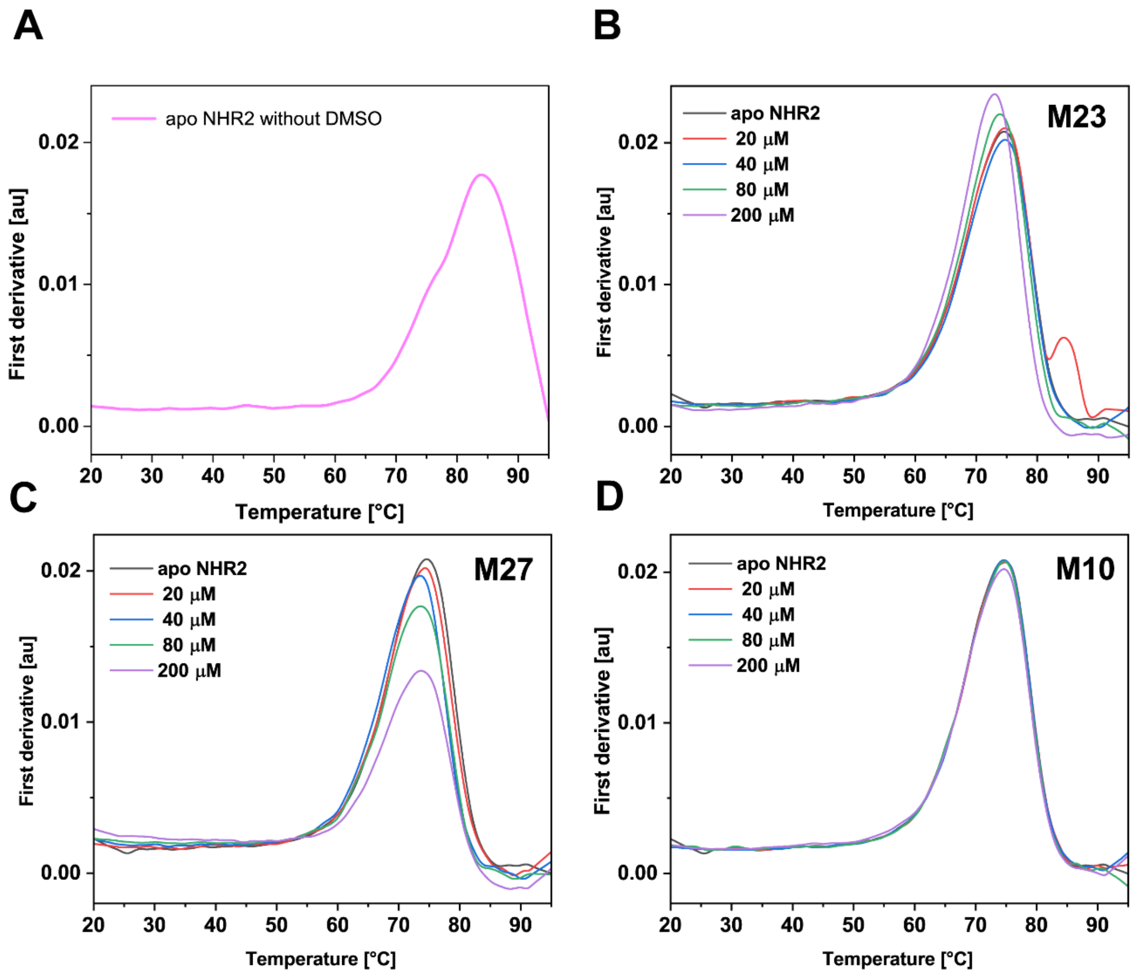

Figure S3. Concentration-dependent melting curves of NHR2 measured by nanoDSF. To determine the thermal unfolding transition (T_m) of *apo* NHR2 without 10% of DMSO **(A)** and then in the presence of the hit compounds, samples were prepared by mixing 20 µM of NHR2 and 20, 40, 80, and 200 µM of the respective compound **(B) M23**, **(C) M27**, or **(D) M10** to the final volume of 50 µl in a buffer consisting of 50 mM sodium phosphate, 50 mM sodium chloride, pH 8.0, 10% (v/v) DMSO. Changes in the ratio of fluorescence emission at 350 nm / 330 nm indicate blue- or redshifts. The first-order derivatives of the thermal unfolding events are shown. A T_m of 84.1 ± 0.15 °C (panel A) and 74.5 ± 0.16 °C (panels B-D) were observed for *apo* NHR2 without and with 10% of DMSO, and the presence of the compounds lowered the T_m with increasing compound concentration except for **M10**.

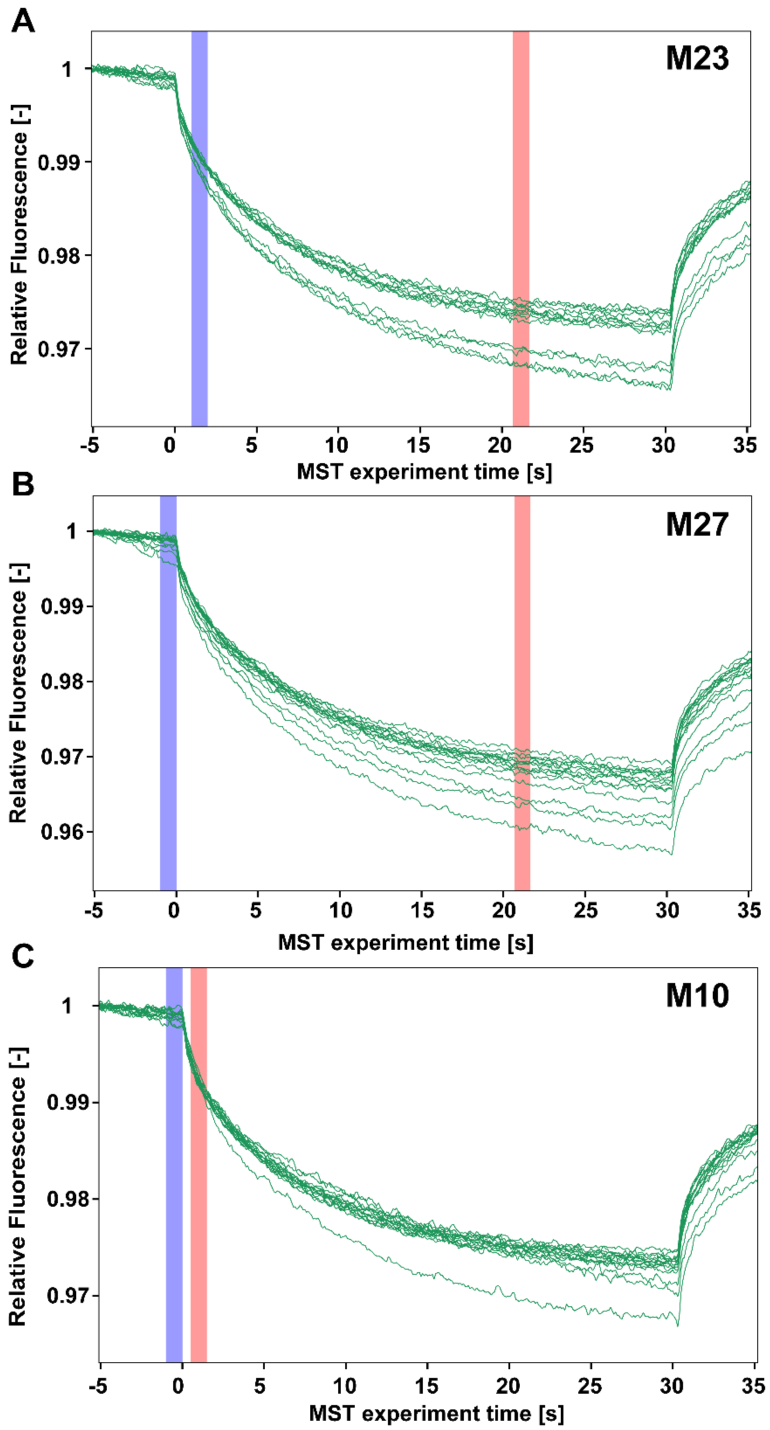

Figure S4. MST traces of the binding of the inhibitor compounds to NHR2. Hit compounds, **(A) M23**, **(B) M27**, **(C) M10,** binding to NHR2 detected by MST assay is shown. Titration of compounds to a constant concentration of Alexa488 dye-labeled NHR2 induces a change in thermophoresis. Regions of the traces corresponding to T-jump and thermophoresis signals were used (blue and red bars) for obtaining apparent K_D values, that is, **M23** was analyzed by thermophoresis, **M27** was analyzed by T-jump and thermophoresis, and **M10** was analyzed by T-jump. These methods were chosen based on the response amplitude and/or standard error of the MST traces.

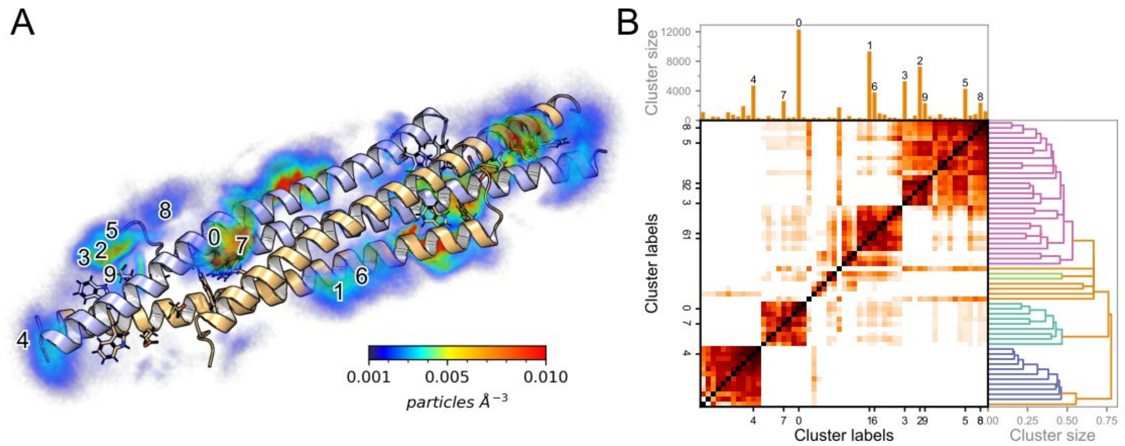

Figure S5. Binding of M23 to the NHR2 tetramer. (A) Heavy-atom particle density of **M23** around the NHR2 tetramer is mapped in a color-coded manner (see color scale). A clustering of ligand poses was performed based on interaction fingerprints with NHR2 residues. **(B)** Similarity between the clusters is shown for all clusters with a population > 0.1% of the total number of samples. Clusters with a population > 1.0% are labeled in the order of decreasing population: 0 (largest) to 9 (smallest).

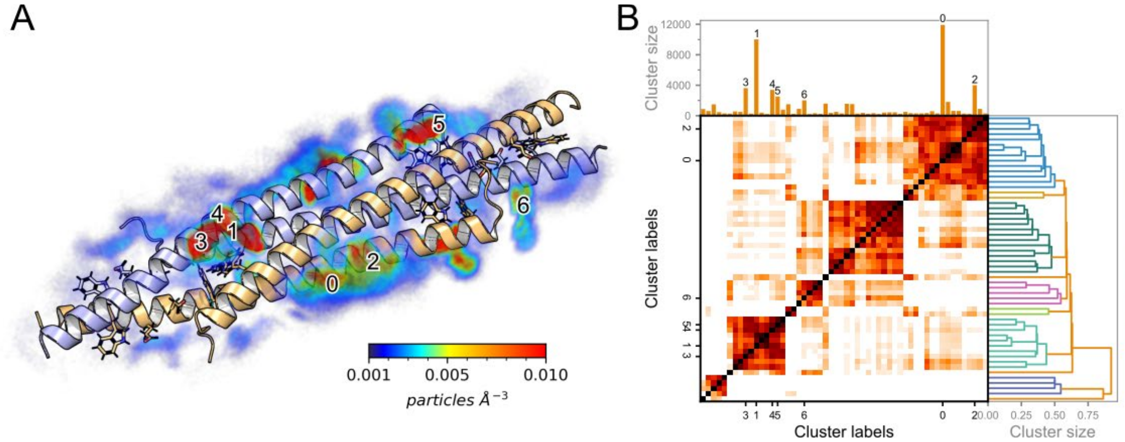

Figure S6. Binding of M27 to the NHR2 tetramer. (A) Heavy-atom particle density of **M27** around the NHR2 tetramer is mapped in a color-coded manner (see color scale). A clustering of ligand poses was performed based on interaction fingerprints with NHR2 residues. **(B)** Similarity between the clusters is shown for all clusters with a population > 0.1% of the total number of samples. Clusters with a population > 1.0% are labeled in the order of decreasing population: 0 (largest) to 6 (smallest).

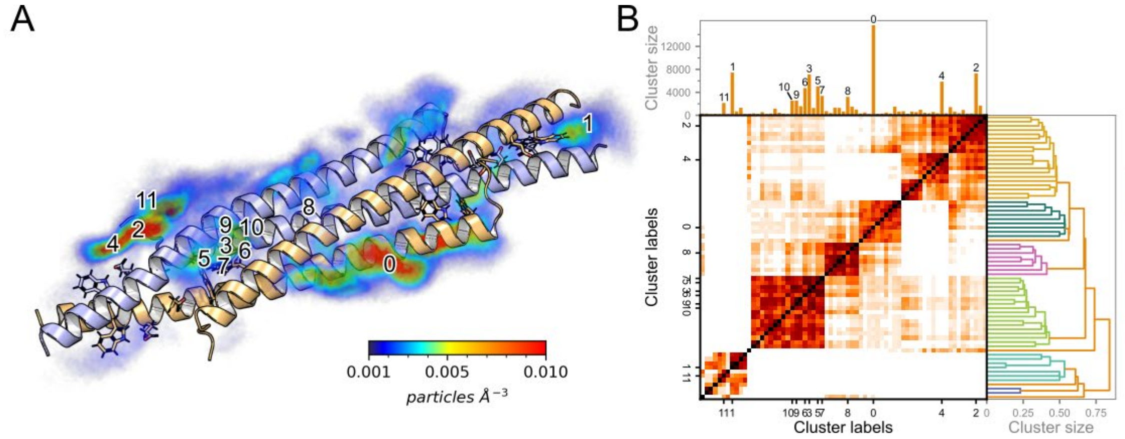

Figure S7. Binding of M10 to the NHR2 tetramer. (A) Heavy-atom particle density of **M10** around the NHR2 tetramer is mapped in a color-coded manner (see color scale). A clustering of ligand poses was performed based on interaction fingerprints with NHR2 residues. **(B)** Similarity between the clusters is shown for all clusters with a population > 0.1% of the total number of samples. Clusters with a population > 1.0% are labeled in the order of decreasing population: 0 (largest) to 10 (smallest).

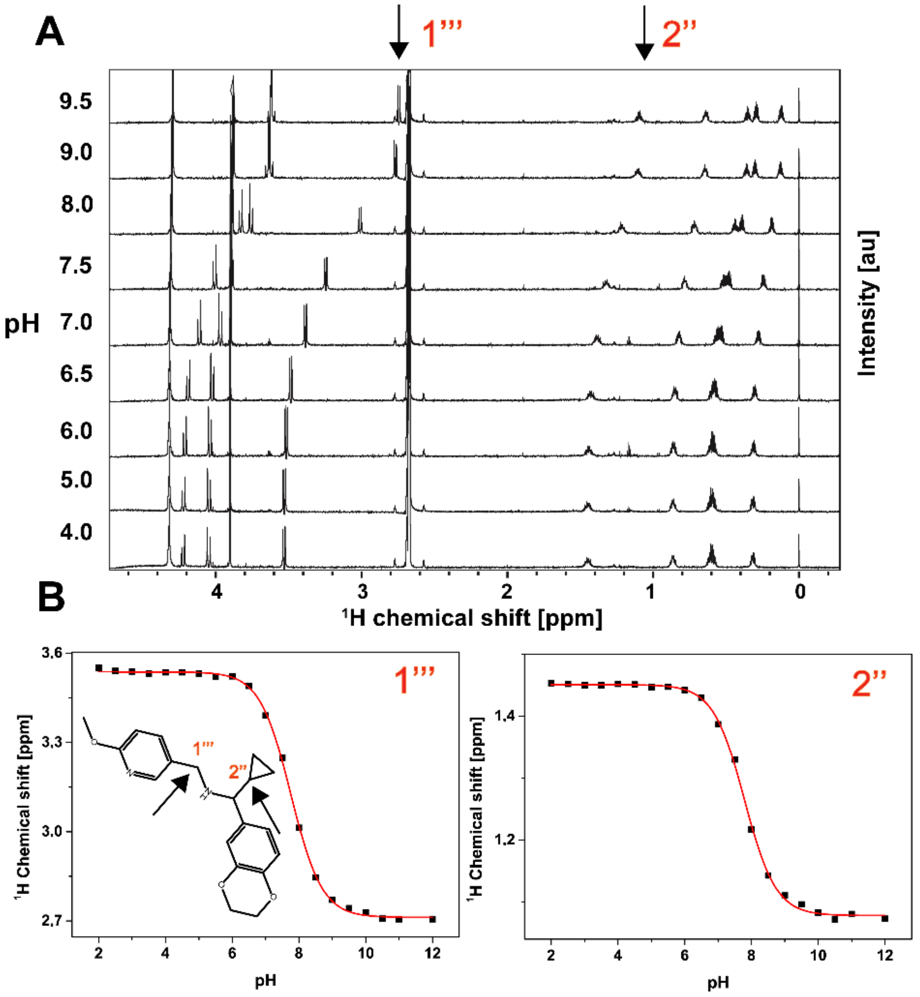

Figure S8. Determination of the pK_a value of M27 by NMR. (**A**) Resolved high-field portion of the 700 MHz ^{1}H NMR spectrum of 300 µM **M27** measured in the pH range of 2 to 13 in 50 mM sodium phosphate, 100 mM sodium chloride, 10% (v/v) D_2O, 10% (v/v) DMSO-d_6. Sodium 2,2-dimethyl-2-silapentane-5-sulfonate (DSS) was used for chemical shift referencing and calibrated to a zero ppm value. (**B**) Chemical shift values of reporter protons (labeled as 1''' and 2'', marked by arrows) were plotted against pH. The pK_a value was calculated by fitting to the Henderson-Hasselbalch equation. The pK_a value of the secondary amine is 7.76 ± 0.01 and 7.79 ± 0.02 as indicated by protons 1''' and 2'', respectively.

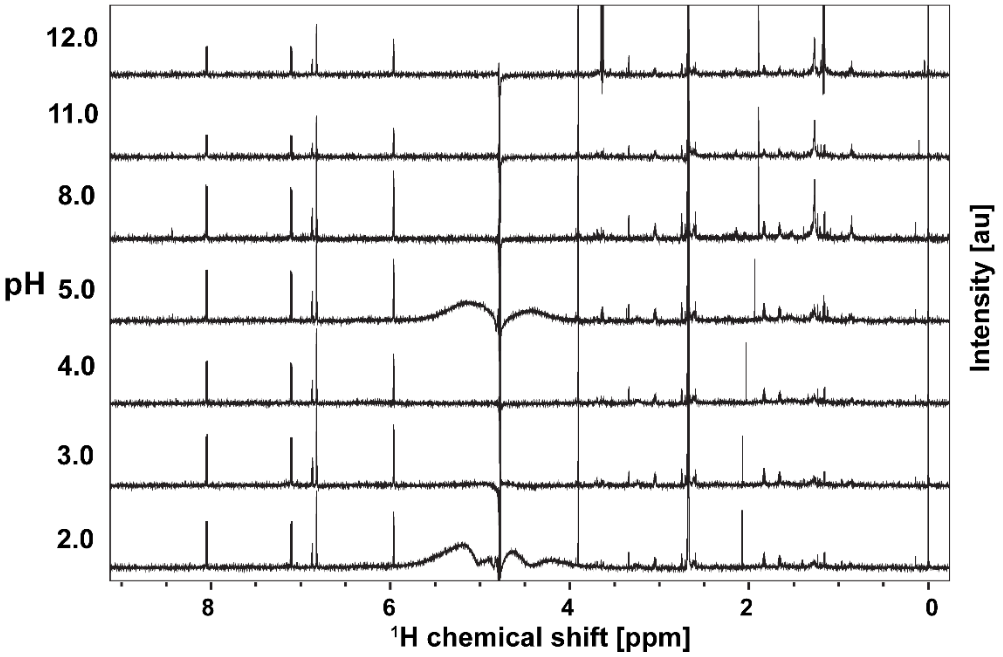

59
60 **Figure S9. M10** does not contain a labile proton and did not show a chemical shift change during pH titration.

Supplementary tables

Table S1. Molecules selected from ligand-based virtual screening for experimental testing. Molecules with an IC_{50} value < 1 mM in the cell viability assay and a positive signal in the STD NMR experiment are marked in green.

Compound ID	ZINC ID[a]	MolPort ID[a]	Company[b]	Catalog ID[b]	MW[c]	xlogP[d]
**M1**	ZINC2698109	MolPort-000-479-588	Life Chemicals Inc	F1533-0315	292	1.12
**M2**	ZINC4279993	MolPort-000-466-718	Life Chemicals Inc	F1278-0182	322	2.51
**M3**	ZINC13628649	MolPort-000-842-332	Eximed	EiM07-18223	374	3.34
**M4**	ZINC4125019	MolPort-002-125-097	ChemBridge Corporation	7731187	272	2.40
**M5**	ZINC299739741	MolPort-039-279-427	Enamine Ltd	Z1603366153	350	2.53
**M6**	ZINC238012695	MolPort-039-278-948	Enamine Ltd	Z929743448	329	2.71
**M7**	ZINC1397593	MolPort-001-843-075	BIONET/Key Organics Ltd	7J-537S	302	3.28
**M8**	ZINC4107100	MolPort-002-886-926	BIONET/Key Organics Ltd	MS-2031	330	3.08
**M9**	ZINC49882203	Not available in MolPort database	Sigma Aldrich	PH006100	284	3.14
**M10**	ZINC1389510	MolPort-002-869-018	BIONET/Key Organics Ltd	4N-031	314	3.41
**M11**	ZINC2549649	MolPort-006-755-951	BIONET/Key Organics Ltd	MS-6536	351	4.02
**M12**	ZINC13187875	MolPort-000-183-134	Enamine Ltd	Z85969754	357	2.41
**M13**	ZINC8312526	MolPort-004-185-300	Enamine Ltd	Z85881800	340	2.66
**M14**	ZINC13257945	MolPort-005-386-809	Enamine Ltd	Z224125632	359	2.93
**M15**	ZINC8274274	MolPort-005-517-016	Enamine Ltd	Z85881817	352	2.32
**M16**	ZINC42338549	MolPort-009-327-813	Enamine Ltd	Z477284616	373	3.03
**M17**	ZINC97035318	MolPort-029-929-656	Enamine Ltd	Z1603203274	338	3.32
**M18**	ZINC5344491	MolPort-002-877-145	BIONET/Key Organics Ltd	7J-549S	306	3.92
**M19**	ZINC1397637	MolPort-002-877-148	BIONET/Key Organics Ltd	7J-571S	302	4.09
**M20**	ZINC216966461	MolPort-035-759-722	ChemBridge Corporation	16303929	333	3.10
**M21**	ZINC95384280	MolPort-028-594-163	ChemBridge Corporation	81622110	371	2.72
**M22**	ZINC32759522	MolPort-009-382-435	Enamine Ltd	Z106335344	371	3.01
**M23**	ZINC9994366	MolPort-004-217-884	Enamine Ltd	Z106912176	399	3.71
**M24**	ZINC58405221	MolPort-009-590-667	Enamine Ltd	Z1142473439	314	2.05
**M25**	ZINC89905076	MolPort-027-668-844	Enamine Ltd	Z1492372136	323	3.20
**M26**	ZINC89762015	MolPort-028-820-057	Enamine Ltd	Z152080224	403	2.21
**M27**	ZINC97034212	MolPort-029-929-350	Enamine Ltd	Z1595536589	326	3.10
**M28**	ZINC170071403	MolPort-030-014-742	Enamine Ltd	Z1624129719	329	3.12
**M29**	ZINC22703670	MolPort-006-867-651	Enamine Ltd	Z18362869	317	1.82
**M30**	ZINC328594644	MolPort-039-294-016	Enamine Ltd	Z1839596229	343	2.73

[a] The ZINC ID and MolPort ID of the stereoisomers based on which the compound was chosen for the experimental testing.
[b] All 30 compounds purchased from the vendors are listed along with their catalog numbers at the time of purchase. The actual configuration of the compounds was not redetermined by us. 12 compounds tested in more detail are highlighted in green.
[c], [d] The molecular weight (MW) and xlogP values were taken from the ZINC database.

Table S2. Permeability data of reference compounds from Gopalswamy et al. (1).[a]

**Compound**	**log $P_{\mathrm{eff/PMF}}$ P_0^{-1} [b]**	**log $P_{\mathrm{eff/PAMPA}}$ P_0^{-1} [c]**
progesterone	0.70 ± 0.28	-4.94
theophylline	0.12 ± 0.50	-5.91
pralidoxime (P2-PAM)	-9.71 ± 0.74	-7.52
atropine	-0.75 ± 0.94	-5.26
chlorpromazine	1.58 ± 0.18	-5.26
diazepam	1.10 ± 0.16	-5.40
asoxime (HI-6)	-14.43 ± 0.91	-7.69
methoxime (MMB4)	-10.67 ± 1.32	-9.25
promazine	1.46 ± 0.17	-4.88

[a] Note that for some of the values, typos have been corrected here compared to the respective table in ref. (1). All computations in ref. (1) were done with the correct values.

[b] $P_{\mathrm{eff/PMF}}$, effective permeability in cm sec^{-1} calculated from free energy calculations (eq. S2). P_0, unit factor corresponding to 1 cm sec^{-1}. Errors correspond to the standard deviation obtained from calculating the permeability when dividing 50 ns into ten independent 5 ns simulation slices.

[c] $P_{\mathrm{eff/PAMPA}}$, effective permeability in cm sec^{-1} obtained from PAMPA assays (2). P_0, unit factor corresponding to 1 cm sec^{-1}.

Supplementary methods

Permeability estimation from molecular dynamics simulations

Molecular dynamics simulations

For setting up molecular dynamics (MD) simulations, atom types and their corresponding parameters were obtained from the AMBER GAFF2 force field (3), using antechamber. The restrained electrostatic potential (RESP) method was used to assign partial charges from electrostatic potentials calculated at the HF/6-31G* level of theory with Gaussian 09 (4). Each simulation system was packed in a 75x75 $Å^2$ 1,2-dioleoyl-*sn*-glycero-3-phosphocholine (DOPC) bilayer plane within a 100 Å tall simulation box using PACKMOL-Memgen (5), using TIP3P water molecules for the water phase (6), and one ligand in the center of the bilayer. Cl^- ions were added to neutralize the system when positively charged compounds were simulated.

All MD simulations were performed using AMBER22 (3). The minimization of the systems was performed using the MPI implementation of PMEMD (7). 5000 cycles of steepest descent were followed in the first step of minimization by conjugate gradient minimization for a total of 10000 cycles. Only water molecules (and ions, if included) were minimized initially, using harmonic restraints of 25 kcal mol^{-1} A^{-2} on the rest of the system. In the second minimization step, the harmonic restraints were decreased to 5 kcal mol^{-1} A^{-2}, while in the third and fourth steps of minimization, the restraints were applied only on the ligand. The fifth minimization step was performed without restraints. The minimized systems were then heated from 0 to 100 K for 5 ps in the NVT ensemble. The temperature was controlled using Langevin dynamics with a coupling constant of 1 ps^{-1}, SHAKE, and a time step of 2 fs in all cases (8). Further heating to 300 K was performed under NPT conditions, using the Berendsen barostat with semiisotropic pressure scaling along the membrane plane for 115 ps. The simulations were further relaxed under the same conditions until 5 ns were obtained, keeping the compound molecules at the center of the membrane bilayer with a harmonic restraint along the membrane normal (z-axis) of 2.5 kcal mol^{-1} A^{-2}.

The permeabilities of the selected compounds were calculated in a similar fashion as done by us and elsewhere (1,9). In brief, steered MD simulations were carried out for each molecule, pulling for 32 ns at 300 K with a force constant of 10 kcal mol^{-1} A^{-2} and a pulling speed of 1 Å ns^{-1} along the membrane normal (z-axis). A total of 33 umbrella windows were extracted, covering 0 to 32 Å with respect to the membrane center along the z-axis. In umbrella sampling simulations, each extracted structure was simulated for 100 ns, maintaining as a reaction coordinate the distance to the membrane center along the z-axis in the umbrella window with a

force constant of 2.5 kcal mol^{-1} Å^{-2}. The initial 50 ns of each simulation were considered as equilibration time, with the last 50 ns of each simulation being used for further analysis.

Permeation potential of mean force and permeability calculations

Potential of mean force (PMF) profiles were calculated from the distance distributions obtained from the umbrella sampling simulations, using the weighted histogram analysis method (WHAM) (10). A value of zero was assigned for the molecule in bulk water (**Figure 8A**). The permeability of the compounds was calculated following the same protocol as done by us before (1). Briefly, as described by Hummer (11), the resistivity (R) to the permeation through the membrane of the compounds (eq. S1) can be computed by using the PMF profile ($\Delta G(z)$) and the diffusion along the membrane normal (z-axis) ($D(z)$) (12,13):

$$R(z) = \frac{e^{\beta(\Delta G(z))}}{D(z)} \qquad \text{(S1)}$$

where β is the inverse of the Boltzmann constant times the absolute temperature, $D\big(z = (z)\big) = \frac{var(z)}{\tau_z}$ and τ_z corresponds to the characteristic time of the z-position autocorrelation in the given window. The inverse of the integral of the resistivity (R) along the z-axis (eq. S2) results in the effective permeability P_{eff} (12) (eq. S2):

$$P_{eff} = \frac{1}{R} = \frac{1}{\int_0^z R(z)\,dz} \qquad \text{(S2)}$$

where the range 0 to z covers the width of the whole membrane (2,14). Errors of the calculated permeabilities were estimated by performing the calculations in ten slices of 5 ns from the total 50 ns used for the analysis.

The calibration curve obtained previously (1) for the compounds with experimentally determined PAMPA data of the reference molecules by Bennion et al. (2) (log $P_{eff/PMF}$ P_0^{-1} and log $P_{eff/PAMPA}$ P_0^{-1}, respectively, **Table S2, Figure 8B**) was used to calculate PAMPA permeabilities for the compounds in **Table 2**.

Supplementary references

1. Gopalswamy, M., Kroeger, T., Bickel, D., Frieg, B., Akter, S., Schott-Verdugo, S., Viegas, A., Pauly, T., Mayer, M., Przibilla, J., Reiners, J., Nagel-Steger, L., Smits, S. H. J., Groth, G., Etzkorn, M., and Gohlke, H. (2022) Biophysical and pharmacokinetic characterization of a small-molecule inhibitor of RUNX1/ETO tetramerization with anti-leukemic effects. *Scientific Reports* **12**
2. Bennion, B. J., Be, N. A., McNerney, M. W., Lao, V., Carlson, E. M., Valdez, C. A., Malfatti, M. A., Enright, H. A., Nguyen, T. H., Lightstone, F. C., and Carpenter, T. S. (2017) Predicting a Drug's Membrane Permeability: A Computational Model Validated With in Vitro Permeability Assay Data. *J Phys Chem B* **121**, 5228-5237
3. D.A. Case, K. B., I.Y. Ben-Shalom, S.R. Brozell, D.S. Cerutti, T.E. Cheatham, III, V.W.D. Cruzeiro, T.A. Darden, R.E. Duke, G. Giambasu, M.K. Gilson, H. Gohlke, A.W. Goetz, R. Harris, S. Izadi, S.A. Izmailov, K. Kasavajhala, A. Kovalenko, R. Krasny, T. Kurtzman, T.S. Lee, S. LeGrand, P. Li, C. Lin, J. Liu, T. Luchko, R. Luo, V. Man, K.M. Merz, Y. Miao, O. Mikhailovskii, G. Monard, H. Nguyen, A. Onufriev, F.Pan, S. Pantano, R. Qi, D.R. Roe, A. Roitberg, C. Sagui, S. Schott-Verdugo, J. Shen, C.L. Simmerling, N.R.Skrynnikov, J. Smith, J. Swails, R.C. Walker, J. Wang, L. Wilson, R.M. Wolf, X. Wu, Y. Xiong, Y. Xue, D.M. York and P.A. Kollman. (2020) AMBER 2020; University of California, San Francisco, CA https://ambermd.org/AmberMD.php. **2020 oct 5**
4. Cornell, W. D., Cieplak, P., Bayly, C. I., and Kollman, P. A. (1993) Application of Resp Charges to Calculate Conformational Energies, Hydrogen-Bond Energies, and Free-Energies of Solvation. *J. Am. Chem. Soc.* **115**, 9620-9631
5. Schott-Verdugo, S., and Gohlke, H. (2019) PACKMOL-Memgen: A Simple-To-Use, Generalized Workflow for Membrane-Protein-Lipid-Bilayer System Building. *J Chem Inf Model* **59**, 2522-2528
6. Price, D. J., and Brooks, C. L. (2004) A modified TIP3P water potential for simulation with Ewald summation. *J. Chem. Phys.* **121**, 10096-10103
7. Le Grand, S., Gotz, A. W., and Walker, R. C. (2013) SPFP: Speed without compromise-A mixed precision model for GPU accelerated molecular dynamics simulations. *Comput. Phys. Commun.* **184**, 374-380
8. Miyamoto, S., and Kollman, P. A. (1992) Settle - an Analytical Version of the Shake and Rattle Algorithm for Rigid Water Models. *J. Comput. Chem.* **13**, 952-962
9. Bennion, B. J., Be, N. A., McNerney, M. W., Lao, V., Carlson, E. M., Valdez, C. A., Malfatti, M. A., Enright, H. A., Nguyen, T. H., Lightstone, F. C., and Carpenter, T. S. (2017) Predicting a Drug's Membrane Permeability: A Computational Model Validated With in Vitro Permeability Assay Data. *J Phys Chem B* **121**, 5228-5237.
10. Kumar, S., Bouzida, D., Swendsen, R. H., Kollman, P. A., and Rosenberg, J. M. (1992) The Weighted Histogram Analysis Method for Free-Energy Calculations on Biomolecules .1. The Method. *J. Comput. Chem.* **13**, 1011-1021
11. Hummer, G. (2005) Position-dependent diffusion coefficients and free energies from Bayesian analysis of equilibrium and replica molecular dynamics simulations. *New Journal of Physics* **7**, 34-34
12. Lee, C. T., Comer, J., Herndon, C., Leung, N., Pavlova, A., Swift, R. V., Tung, C., Rowley, C. N., Amaro, R. E., Chipot, C., Wang, Y., and Gumbart, J. C. (2016) Simulation-Based Approaches for Determining Membrane Permeability of Small Compounds. *J Chem Inf Model* **56**, 721-733
13. Dickson, C. J. (2020) AMBER-Umbrella_COM_restraint_tutorial. *https://github.com/callumjd/AMBER-Umbrella_COM_restraint_tutorial* **2020 oct 08**

14. Carpenter, T. S., Kirshner, D. A., Lau, E. Y., Wong, S. E., Nilmeier, J. P., and Lightstone, F. C. (2014) A method to predict blood-brain barrier permeability of drug-like compounds using molecular dynamics simulations. *Biophys J* **107**, 630-641
